# Sponges Lack ParaHox Genes

**DOI:** 10.1093/gbe/evz052

**Published:** 2019-03-11

**Authors:** Claudia C Pastrana, Melissa B DeBiasse, Joseph F Ryan

**Affiliations:** 1 Whitney Laboratory for Marine Bioscience, University of Florida, St. Augustine; 2 Department of Biology, University of Miami; 3 Department of Biology, University of Florida

**Keywords:** Cdx, Hox, ParaHox, Porifera, sponge

## Abstract

Addressing the origin of axial-patterning machinery is essential for understanding the evolution of animal form. Historically, sponges, a lineage that branched off early in animal evolution, were thought to lack Hox and ParaHox genes, suggesting that these critical axial-patterning genes arose after sponges diverged. However, a recent study has challenged this long-held doctrine by claiming to identify ParaHox genes (*Cdx* family) in two calcareous sponge species, *Sycon ciliatum* and *Leucosolenia complicata*. We reanalyzed the main data sets in this paper and analyzed an additional data set that expanded the number of bilaterians represented and removed outgroup homeodomains. As in the previous study, our Neighbor-Joining analyses of the original data sets recovered a clade that included sponge and *Cdx* genes, whereas Bayesian analyses placed these sponge genes within the NKL subclass of homeodomains. Unlike the original study, only one of our two maximum-likelihood analyses was congruent with *Cdx* genes in sponges. Our analyses of our additional data set led to the sponge genes consistently being placed within the NKL subclass of homeodomains regardless of method or model. Our results show more support for these sponge genes belonging to the NKL subclass, and therefore imply that Hox and ParaHox genes arose after Porifera diverged from the rest of animals.

## Introduction

Addressing the mechanistic origins of axial-patterning processes of modern animals is essential for a broader understanding of the evolution of animal form. Hox and ParaHox genes are widely recognized as playing a pivotal role in patterning the primary body axis of most animals (Slack et al. 1993; Carroll 2005), but how and when these transcription factors arose is not well understood. As one of the first lineages to branch away from other animals, sponges provide important insight into the early evolution of the developmental toolkit (Degnan et al. 2009), which is critical for understanding the evolution of primary body axes in animals (Ryan and Baxevanis 2007).

Homeobox genes are a large set of highly conserved transcription factors present in the vast majority of eukaryotic lineages (Duboule 1995; de Mendoza et al. 2013). Hox and ParaHox genes, along with Hox-like genes (*Evx, Meox, Mnx, Gbx*), make up the HOXL subclass of the ANTP class of homeoboxes (Holland et al. 2007). The NKL subclass makes up the rest of the ANTP class, one of the 11 classes of the homeobox superfamily (Holland et al. 2007). Hox and ParaHox genes have been identified in almost all animal lineages, but have not been identified in Ctenophora (comb jellies; Ryan et al. 2013) or, until recently, Porifera (sponges; Larroux et al. 2007; Srivastava et al. 2010). Given that Ctenophora and Porifera successively branched off from the rest of animals very early in animal evolution (e.g., Dunn et al. 2008; Ryan et al. 2013; Shen et al. 2017; Simion et al. 2017; Whelan et al. 2017), it was thought that Hox and ParaHox genes arose in the stem ancestor of Parahoxozoa (a clade consisting of Placozoa, Cnidaria, and Bilateria; Ryan et al. 2010).

**Fig. 1.**
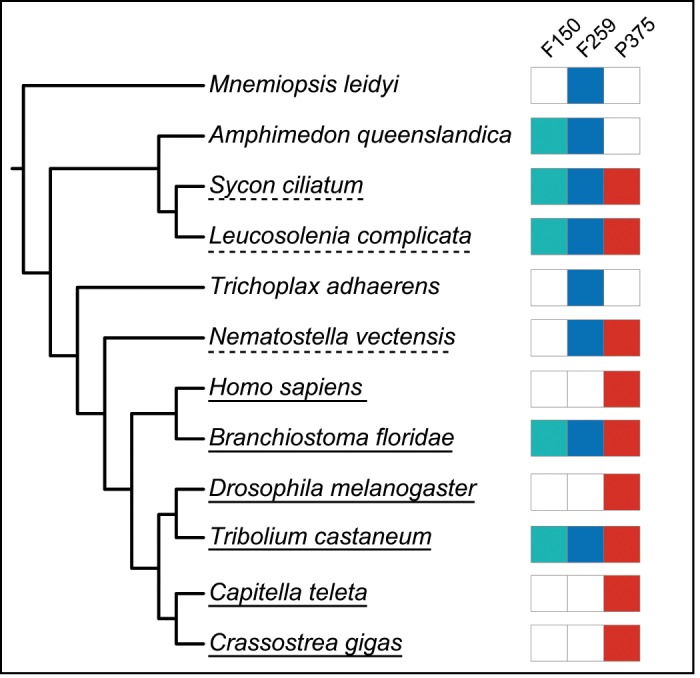
—Taxon sampling for homeodomain data sets in this study. Green boxes represent taxa sampled for the Fortunato et al. 150 homeodomain data set (F150). Blue boxes represent taxa sampled for the Fortunato et al. 259 homeodomain data set (F259). Red boxes represent taxa sampled for the alternative 375 homeodomain data set generated for this study (P375). Taxa with confirmed *Cdx* homeodomains are indicated with a solid underline, taxa with unconfirmed *Cdx* homeodomains are indicated with a dashed underline, and taxa that lack *Cdx* homeodomains are not underlined.

Recently, Fortunato et al. (2014) reported to have identified a ParaHox gene (*Cdx*) in the calcareous sponges *Sycon ciliatum* and *Leucosolenia complicata.* The evidence supporting this claim was not robust to the method of inference (i.e., maximum-likelihood [ML], Neighbor-Joining, and Bayesian methods applied to the same data set did not place these sponge genes in the same clade). This is problematic given that the robustness of methods is an indicator of the phylogenetic signal in a data set and the adequacy of that signal to determine the true phylogeny (Penny et al. 1992). Another concern was that though the results produced a clade containing bilaterian *Cdx* and sponge candidate *Cdx* genes, this clade fell outside of the larger Hox/ParaHox clade (Fortunato et al. 2014; fig. 1; Extended fig. 1). This is unusual as most studies recover monophyletic Hox and ParaHox clades (e.g., Banerjee-Basu and Baxevanis 2001; Chiori et al. 2009; Zwarycz et al. 2015). Interestingly, in examples where Hox/Parahox is not monophyletic, it is often because a nonHox/ParaHox gene is placed in a clade with *Cdx* genes (Holland et al. 2007; Takatori et al. 2008) (e.g., Holland et al. 2007; Takatori et al. 2008), but not always (Larroux et al. 2007).

Several factors may have contributed to the lack of robustness in the results supporting *Cdx* genes in sponges, such as the absence of Spiralia, which make up a large proportion of the diversity within Bilateria. Another potentially confounding issue is the use of previously undescribed short motifs to subsample the main data sets for additional analyses. These subsampled data sets contained only homeodomains that included two motifs named YIS and YIT (three amino-acids starting at position 25 in the homeodomain; Fortunato et al. 2014). This criterion led to paraphyletic sampling of homeodomain families given that *Cdx* was the only HOXL subclass gene in these data sets (Fortunato et al. 2014; Extended figs. 3–5). Sampling based on the YIT/YIS motifs also resulted in the exclusion of the Ankx homeodomain family, with which the putative sponge *Cdx* genes formed a clade in the reported Bayesian analyses (Fortunato et al. 2014). However, because the YIS and YIT motifs have not been previously described in the literature, it is unclear whether their usage to construct alternative data sets is justified.

The ghost locus hypothesis was used as auxiliary support for the claims of a *Cdx* gene in sponges (Fortunato et al. 2014). The ghost locus hypothesis asserts that if in a genomic locus devoid of Hox, there exists a significant number of nonHox genes with bilaterian orthologs in close proximity to Hox clusters, then this locus once contained Hox genes that were subsequently lost (Ramos et al. 2012). The ghost locus hypothesis also applies to ParaHox genes and loci as well. Fortunato et al. (2014) showed that the *S.ciliatumCdx* candidate is in the “neighborhood” of four genes that are orthologous to genes linked to ParaHox loci in humans. However, given that only 14 genes on this scaffold had clear human orthologs, there were insufficient data to test the statistical significance of this hypothesis (Fortunato et al. 2014).

We hypothesize that the finding of a *Cdx* gene in Porifera is sensitive to methods, models, and taxon sampling. To test this hypothesis, we reexamine the data sets from Fortunato et al. (2014) and construct an alternative data set that includes several additional taxa. We analyze all of these data sets using a range of tree-construction methodologies and models.

## Materials and Methods

### Phylotocol, Transparency, and Reproducibility

To maximize transparency and avoid confirmation bias, we constructed a phylotocol (DeBiasse and Ryan 2018), which outlined our planned phylogenetic analyses prior to the start of the project (supplementary file 1, Supplementary Material online). For complete transparency, this document was published on GitHub before the analyses began (May 28, 2017). We followed the protocol as outlined, and made six minor adjustments that we justified and publicly documented prior to executing the proposed changes. The phylotocol, alignments, trees, and commands used in these analyses are available at: https://github.com/josephryan/2018-Pastrana_etal_SpongeParaHoxAnalyses (last accessed February 6, 2019).

### Repeating and Expanding Analyses on Original Data Sets

We repeated the analyses as performed in Fortunato et al. (2014) using two of their original data sets. The first data set contained 150 homeodomains and was used to infer Fortunato’s fig. 1; we refer to this data set as F150 (fig. 1). The second data set contained 259 homeodomains and was used to infer the tree in Fortunato’s Extended fig. 1; we refer to this data set as F259 (fig. 1). These data sets are available as FASTA files in the supplementary information, Supplementary Material online of this paper and at the GitHub link above. We used Prottest v3.0 (Abascal et al. 2005) to confirm choice of model for the F150 and F259 data sets and then performed NJ analyses with Phylip v3.696 (Felsenstein 1993), ML analyses with PhyML v3.0 (Guindon et al. 2010), and Bayesian analyses with MrBayes v3.3.6 (Ronquist et al. 2012).

For new analyses of the original data sets, we performed NJ analyses using Phylip v3.696 with the following models: JTT, PMB, PAM, and Kimura, as implemented in the Protdist program. We performed ML analyses using RAxML v8.2.10 (Stamatakis 2014) under the following models: PROTGAMMALG, PROTGAMMAJTT, PROTGAMMAWAG, and PROTGAMMAAUTO with 100 bootstraps. We chose RAxML over PhyML, which was used in Fortunato et al. (2014), for these new analyses based on reports of their accuracy in a recent review of ML methods (see fig. 2 of Zhou et al. 2018). For these ML analyses, we used five starting parsimony trees and five random starting trees and chose the one with the highest likelihood as determined by RAxML. In all cases, the likelihood values of our best RAxML trees were higher than our PhyML trees. We conducted Bayesian analyses using MrBayes v3.3.6 under the following models: LG, WAG, JTT, and MIXED with gamma-distributed rates across sites.

**Fig. 2.**
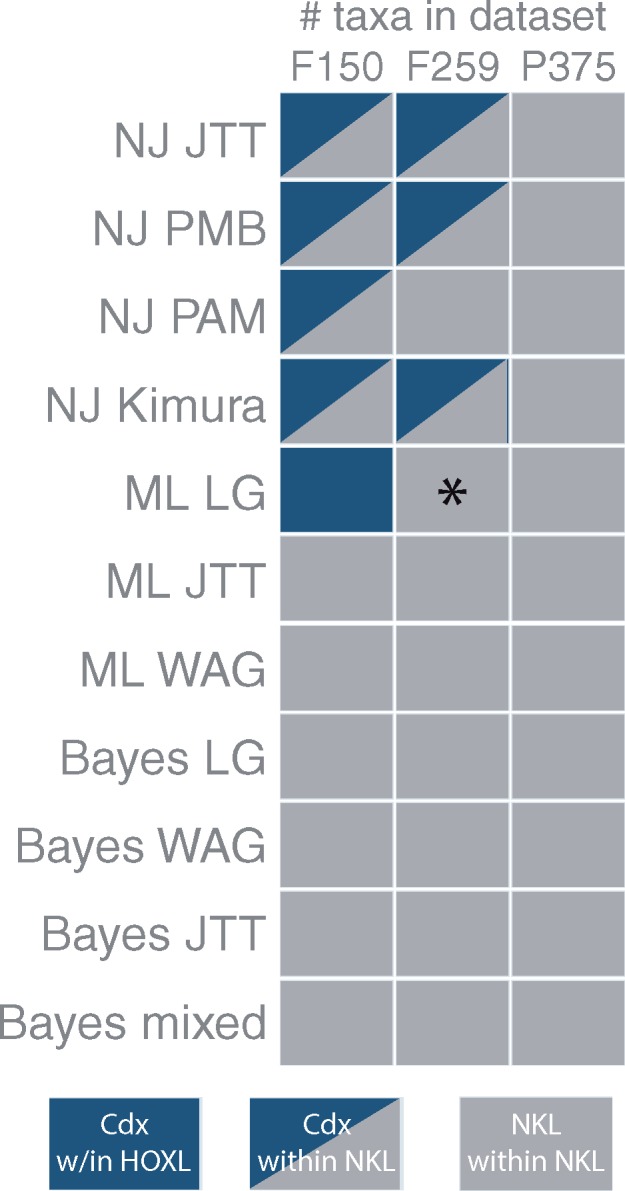
—Support for three different hypotheses regarding sponge candidate *Cdx* genes. The row names indicate the methodology (NJ, ML, or Bayesian) followed by the model (JTT, PAM, PMB, Kimura, LG, WAG, or mixed). Blue boxes indicate that the sponge candidate *Cdx* genes occurred in a clade that included bilaterian *Cdx* genes and that this clade was a subclade of a monophyletic Hox/ParaHox clade. Blue/grey boxes indicate that the sponge candidate *Cdx* genes occurred in a clade that included bilaterian *Cdx* genes, but that this clade was nested within a clade of NKL genes. Grey boxes indicate that the sponge candidate *Cdx* genes occurred in a clade with NKL genes outside of a monophyletic Hox/ParaHox clade. NJ, Neighbor-Joining, ML, maximum likelihood. *Note: this result contrasts with the ML LG analysis of the F259 data set reported in Fortunato et al. (2014); details of these differences are in the Materials and Methods, Results, and Discussion.

### Expanding Taxon Sampling

We constructed an alternative 375-homeodomain data set (referred to as P375; fig. 1 and supplementary table S1, Supplementary Material online). We used HomeoDB (Zhong and Holland 2011) to obtain the complete set of HOXL and NKL sequences for human (*Homo sapiens*), beetle (*Tribolium castaneum*), amphioxus (*Branchiostoma floridae*), and fruitfly (*Drosphila melanogaster*). We assembled the complete set of HOXL and NKL homeodomains for the marine polychaete *Capitella teleta*, the Pacfic oyster *Crassostrea gigas*, and the starlet anemone *Nematostella vectenis* from Zwarycz et al. (2015). Finally, we included the putative *Cdx* genes for *S.ciliatum* and *L.complicata* from Fortunato et al. (2014). This data set is available as a FASTA file in the supplementary information, Supplementary Material online of this paper and at the GitHub link above. We performed NJ, ML, and Bayesian analyses on this alternative data set as described above.

### Hypothesis Testing

We used the approximately unbiased (AU) test (Shimodaira 2002) as implemented in CONSEL v1.20 (Shimodaira and Hasegawa 2001) and the Swofford–Olsen–Waddell–Hillis test (SOWH; Goldman et al. 2000) as implemented in sowhat v0.36 (Church et al. 2015) to compare the following competing hypotheses (table 1): 1) The sponge *Cdx* candidates fall in a clade with Cdx genes ((LcoCdx, SciCdx, BflCdx, TcaCad1, TcaCad2), all other sequences), 2) The sponge *Cdx* candidates fall in a clade with all Hox and ParaHox genes ((all Hox and ParaHox, LcoCdx, SciCdx), all other sequences), and 3) Hox and ParaHox genes form a clade that does not include sponge *Cdx* candidates ((all Hox and ParaHox), LcoCdx, SciCdx, all other sequences). The best-fit model, LG, was used for the AU tests, whereas JTT was used for the SOWH tests, as LG is not available in the current version of sowhat.

**Table 1:** AU Tests Comparing the Best ML Tree under the LG Model for Each Data Set to the Best Tree under the Stated Constraint (Column Headers)

AU Tests			
Data Sets	Hypotheses		
	Sponge Candidate *Cdx* with Bilaterian *Cdx* (Optional Hox/ParaHox Monophyly)	Sponge Candidate *Cdx* with Bilaterian *Cdx* (Required Hox/ParaHox Monophyly	Monophyletic Hox/ParaHox without Sponge Candidate *Cdx* Genes
F150-HD data set	NA	NA	*P *=* *0.490
F259-HD data set	*P *=* *0.453	*P *=* *0.230	NA
P375-HD data set	*P *=* *0.192	*P *=* *0.184	NA

Note.—Each *P*-value can be interpreted as the degree of certainty to which the best tree is more likely than the null hypothesis (column header). NA indicates that the best tree is congruent with the constraint. HD=homeodomain.

### Constrained ML Analysis of the F259 Homeodomain Data Set

We ran a constrained ML analysis of the F259 data set using RAxML v8.2.11. For this analysis we used the “-g” option and introduced a constraint tree that required the bilaterian sequences BflCdx, TcaCad1, TcaCad2 and the sponge sequences LcoCdx, SciCdx to form a clade (this clade was recovered in Extended Data fig. 1 of Fortunato et al. 2014). The “-# 10” option was used to run 10 distinct analyses from 10 separate starting trees. The full command line, constraint tree, and output of this analysis are available at the GitHub link above.

## Results

### Replication of Fortunato et al. (2014) Analyses

**Fig. 3.**
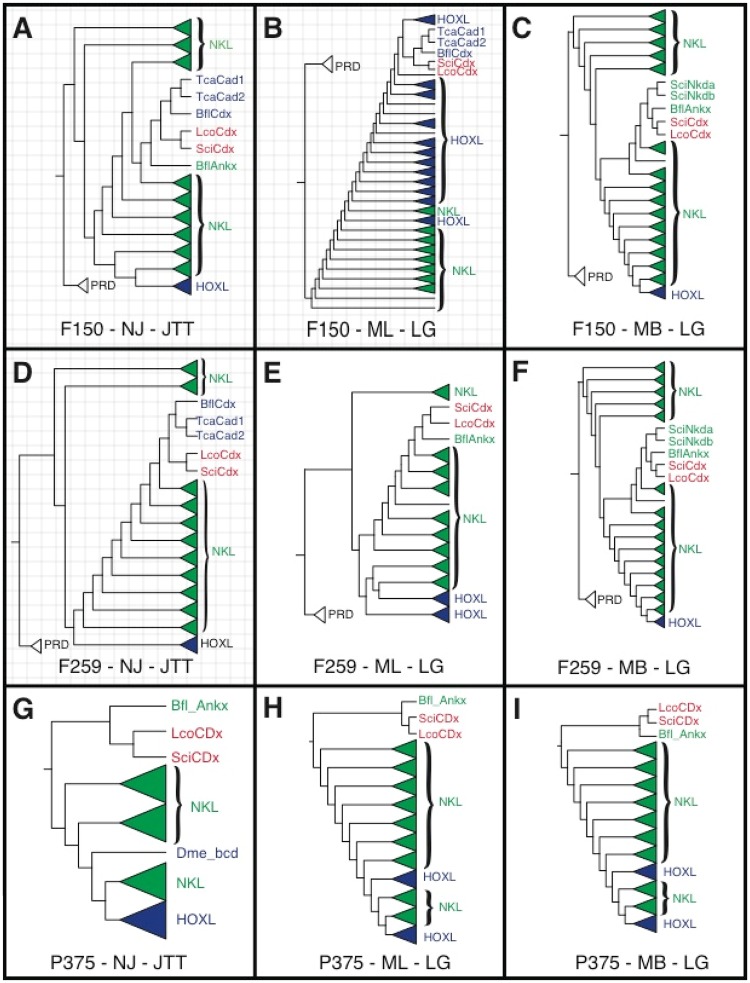
—Summary of phylogenetic analyses for three homeodomain data sets under the optimal substitution model. Each panel includes a dash-delimited code with the first field indicating the data set (F150, F259, or P375), the second field indicating the analysis performed (NJ, ML, or MB), and the third field indicating the optimal model used in the analysis (JTT or LG). Sponge candidate *Cdx* genes are in red, Hox genes are in blue, and NKL genes are in green. Triangles indicate a collapsed clade; the size of the triangle is not indicative of the size of the collapsed clade. Panels with a grid background indicate phylogenies where sponge candidate *Cdx* genes group with bilaterian *Cdx* genes. Support values for all clades are included in the supplement and GitHub. NJ, Neighbor-Joining; ML, maximum likelihood; MB, Bayesian (MrBayes). Bfl, *Branchiostoma floridae*; *Tca, Tribolium castaneum*; Dme, *Drosophila melanogaster*; Lco, *Leucosolenia complicata*; Sci, *Sycon ciliatum*.

The original study (Fortunato et al. 2014) included analyses of two data sets. One consisted of 150 homeodomains (herein called F150) and another consisting of 259 homeodomains (herein called F259). The authors performed the following analyses on the F150 and F259 data sets: 1) NJ with the JTT model, 2) ML with the LG model, and 3) Bayesian with the LG model. We performed the ML analyses in PhyML, as did Fortunato et al. (2014), and in RAxML. The PhyML trees had lower likelihood scores than the trees estimated in RAxML and we therefore report the RAxML trees here and make the PhyML trees available at the GitHub link above. In five of the six Fortunato et al. (2014) analyses that we repeated, we recovered the same results as Fortunato (fig. 2); our NJ analyses of both data sets (fig. 3*A* and *D*) and our ML analysis of the F150 data set (fig. 2*B*) produced a clade that included the sponge candidate *Cdx* genes with the bilaterian *Cdx* genes, whereas our Bayesian analyses of both data sets failed to recover this clade, instead recovering the putative sponge *Cdx* genes in a clade with the *Branchiostoma* Ankx homeodomain within the larger NKL subclass (fig. 3*C* and *F*). Unlike in Fortunato et al. (2014), our ML analyses of the F259 data set did *not* produce a clade that included both sponge and *Cdx* genes (fig. 3*E*). Instead, like the results of our Bayesian analyses, these sponge genes were recovered within a clade that included the *Branchiostoma* Ankx homeodomain within the larger NKL subclass.

We did not have access to the treefile generated from the ML analysis of the F259 data set in Fortunato et al. (2014), so we were unable to compare the likelihood of that tree with our best tree. To test whether a more likely tree with the sponge-*Cdx* clade existed, we conducted ten additional ML analyses of the F259 data set where we constrained the putative sponge *Cdx* genes to form a clade with bilaterian *Cdx* genes. The likelihood score of the best constrained tree (-14657.885041) was suboptimal to the likelihood score of our best unconstrained analysis of the same data set (−14643.142355). An AU-test comparing these two topologies showed that the differences between the topologies were not significant (*P *=* *0.367). The files associated with this analysis are available at the GitHub link above.

### Model Sensitivity

To test whether the results reported in Fortunato et al. (2014) were sensitive to model choice, we ran NJ, ML, and Bayesian analyses of the F150 and F259 data sets under alternative models where fit was suboptimal, but closer to optimal than other available models. As in the analyses with the most optimal model (JTT; fig. 3*A*), NJ analyses of the F150 data set with PAM, PMB and Kimura produced a clade that included both the putative sponge *Cdx* and bilaterian *Cdx* homeodomains, albeit situated within a larger NKL clade, making Hox/ParaHox paraphyletic (supplementary fig. S1, Supplementary Material online). Likewise, as we found in our NJ analyses of the F259 data set under JTT, the PMB and Kimura models produced the same clade of putative sponge *Cdx* and bilaterian *Cdx* homeodomains (supplementary fig. S2, Supplementary Material online). However, the NJ analysis of the F259 data set under PAM produced a clade that included the putative sponge *Cdx* homeodomains with the *Branchiostoma* Ankx homeodomain within the larger NKL subclass (supplementary fig. S2, Supplementary Material online). These results suggest that the NJ analyses of the F259 data set were sensitive to the models that we tested whereas the NJ analyses of the F150 data set were not.

### Phylogenetic Analysis of an Alternative Data Set

As the focus of this study was to test whether *Cdx* genes exist in sponges, it was important to expand the number of taxa that include bona fide *Cdx* genes and less important to include taxa that lacked these genes. Therefore, we created an alternative data set consisting of 375 homeodomains (herein referred to as P375) that unlike the previous study, included homeodomains from *H.sapiens*, *D.melanogaster*, *C.teleta*, and *C.gigas*, and did not include sequences from *Mnemiopsis leidyi* (present in F259), *Amphimedon queenslandica* (present in F150 and F259), or *Trichoplax adhaerens* (present in F259; fig. 1). This set included the two putative *Cdx* genes from *S.ciliatum* and *L.complicata*, but did not include other homeodomains from these sponges. As in the Fortunato et al. (2014) alignments, we included *B.floridae*, *T.castaneum*, and *Nematostella vectensis* (F259 only). Unlike Fortunato et al. (2014), which included PRD-class outgroups for both F150 and F259, this alternative data set consisted of only ANTP-class sequences, as specifying the root of the ANTP class was unnecessary to the goals of our study.

We performed the same NJ, ML, and Bayesian analyses on the P375 data set as were performed on the F150 and F259 data sets and found that this alternative data set produced consistent results as to the position of the sponge candidate *Cdx* homeodomains. In all trees estimated with the P375 data set, the sponge sequences formed a clade with Ankx within the larger NKL subclass clade (fig. 3*G*–*I*, supplementary fig. S3, Supplementary Material online).

### Hypothesis Testing

We used the AU test to compare relevant hypotheses about the placement of sponge putative *Cdx* genes. The three hypotheses we tested were: 1) the sponge candidate *Cdx* genes form a clade with all other *Cdx* genes, 2) the sponge candidate *Cdx* genes form a clade with all other *Cdx* genes inside the Hox/ParaHox clade, and 3) Hox and ParaHox genes form a clade that excludes the sponge candidate *Cdx* genes (table 1). The first two hypotheses have both sponge candidate *Cdx* genes forming a clade with bilaterian *Cdx* genes, but the first is more lenient, not requiring the *Cdx* clade to fall within the greater Hox/ParaHox clade. The third hypothesis is incongruent with the first two hypotheses.

Despite the lack of support in our trees for bona fide sponge *Cdx* homeodomains, our hypothesis tests did not differentiate among alternative hypotheses (table 1). For the F150 data set, the best ML tree under the LG model was congruent with the first two constraints, so we did not conduct AU tests for these constraints. The *P* value of our test comparing the best tree to a monophyletic Hox/ParaHox cluster excluding the sponge candidate *Cdx* genes under the F150 data set was 0.490 (table 1). The best ML tree under the LG model for the F259 and P375 data sets included a monophyletic Hox/ParaHox cluster that excluded sponge candidate *Cdx* genes. When we compared the best topology for the F259 data set to one that includes sponge candidate *Cdx* with bilaterian *Cdx* genes, the *P* value was 0.453. The *P* value when we constrained this clade to the Hox/ParaHox clade was 0.230 (table 1). Under the P375 data set, the *P* value of the sponge candidate *Cdx* with bilaterian *Cdx* was 0.192 when Hox/ParaHox monophyly was optional and was 0.184 when Hox/ParaHox monophyly was enforced. We also generated comparable results using the SOWH test (supplementary table S2, Supplementary Material online). None of these results conclusively rejects the alternative hypotheses that we proposed.

## Discussion

Prior to Fortunato et al. (2014), it was widely accepted that sponges lacked Hox and ParaHox genes. Our re-analyses of the data sets from Fortunato et al. (2014) show that the original results are sensitive to method, model, and taxon sampling. As such, the results are insufficient to support the presence of ParaHox genes in sponges. In contrast, our analyses of an arguably more appropriate data set consistently recover these sponge genes as NKL homeodomains regardless of method or model, suggesting that the P375 data set is not sensitive to the models and methods that we tested. Further, the majority of phylogenetic results, including all but one of the trees from likelihood-based methods, contradicts the conclusions reached in Fortunato et al. (2014). Considered in toto, these results suggest that the sponge *Cdx* candidates belong to the NKL subclass of homeoboxes.

In the majority of our trees, the sponge gene is recovered in a clade with Ankx. To date, Ankx has only been found in branchiostomids (lancelets; Zhong and Holland 2011). It is possible, but difficult to support from a parsimony perspective, that this gene was present in the last common ancestor of sponges and lancelets and lost in all other descendant lineages. Given that the branches leading to Ankx, the bilaterian *Cdx*, and the supposed sponge *Cdx* homeodomains are all amongst the top 10% in terms of length in our trees, a more parsimonious (albeit untested) scenario is that the placement of these sponge genes is an artifact influenced by long-branch attraction.

Phylogenetic relationships inferred from homeodomains are notoriously difficult to resolve due to low nodal support (Holland et al. 2007). The biggest reason for this constraint is the limited number of characters (60 amino acids) in these genes. Often, there is consistency between analyses and strong support for relationships at the level of homeobox family. For example, support for the distalless clade containing homeodomains from *T. adhaerens*, *N. vectensis*, *B. floridae*, *T. castaneum* is 97 in our ML analysis of the F259 data set (supplementary fig. S4, Supplementary Material online). However, relationships between homeobox families are typically poorly supported and inconsistent between analyses, particularly when classifying homeoboxes of nonbilaterians where homeodomains from these animals often have descended from ancestors that gave rise to multiple named homeodomain families in the bilaterian lineage. These challenges are likely involved in the inability of our hypothesis tests to distinguish among alternative topologies.

In an effort to maximize transparency, this study is one of the first to utilize phylotocol (DeBiasse and Ryan 2018). Before to performing any analyses, we planned our experiments a priori and made our plan public on GitHub (https://github.com/josephryan/2017-SpongeParaHoxAnalyses). We made six revisions to this document during the course of the study and documented each of these changes in subsequent versions of the phylotocol (supplementary file 1, Supplementary Material online). Our aim was to avoid making changes based on confirmation bias; we encourage those evaluating this study to examine these changes alongside the “work completed so far” section and judge the merits of our justifications.

## Conclusion

The evidence herein casts substantial doubt on the presence of a direct ortholog of a ParaHox gene in the sponges *S.ciliatum* and *L. complicata*. Our analyses show that the position of the sponge *Cdx* candidate genes reported in Fortunato et al. (2014) are dependent on model, methodology, and taxon-sampling employed. Our most rigorous methodology (ML and Bayesian) and our alternative data set support sponge candidate *Cdx* genes as being NKL genes. As no other Hox or ParaHox gene has been positively identified in sponges or ctenophores, it suggests that ParaHox genes arose in the stem lineage of Parahoxozoa (but see Ramos et al. 2012) and therefore, the patterning of the primary body axis of the earliest animals must have been achieved with a set of genes that did not include Hox and ParaHox genes.
